# Disrupting Mitochondrial Homeostasis via TBC1D15 Protein

**DOI:** 10.3390/cells15191781

**Published:** 2026-09-30

**Authors:** Pengfei Qiang, Yan Li, Shuang Liang, Miaomiao Jiang, Shuoye Yang, Xiwei Zhang

**Affiliations:** College of Biological Engineering, Henan University of Technology, Zhengzhou 450000, China

**Keywords:** mitochondria, lysosomes, TBC1D15, mitochondrial homeostasis

## Abstract

**Highlights:**

**What are the main findings?**

- Subcellular inter-organelle trafficking of TBC1D15, rather than its whole-cell protein abundance, is identified as the critical switch governing mitochondria–lysosome homeostasis and mitochondrial quality control.
- CCCP and high-dose AuNPs modulate TBC1D15 through two divergent regulatory modes: CCCP induces a PINK1-independent biphasic TBC1D15 fluctuation, whereas AuNPs trap TBC1D15 on the mitochondrial outer membrane and block its lysosomal delivery via a PINK1-independent route.

**What are the implications of the main findings?**

- This work resolves the long-standing ambiguity between TBC1D15 total expression level and its subcellular shuttling, establishing a new conceptual framework that prioritizes protein translocation over bulk abundance in organelle quality control.
- High-dose AuNPs can be exploited as a specific pharmacological inhibitor of TBC1D15 trafficking, offering a potential bypass strategy and therapeutic clues for neurodegenerative and metabolic disorders characterized by defective PINK1/Parkin-mediated mitophagy.

**Abstract:**

Background: Mitochondria–lysosome coupling and mitophagy are fundamental to mitochondrial homeostasis, yet the determinants governing this process remain incompletely defined. This study distinguished the regulatory modes of TBC1D15 and established its functional role as a downstream effector in mitochondria-linked autophagy. Methods: PC-12 and HeLa cells were treated with CCCP, gradient-concentrated AuNPs, and CQ, combined with siRNA-mediated PINK1 knockdown. Whole-cell, mitochondrial and lysosomal fractions were analyzed by immunoblotting. Results: Inter-organelle translocation of TBC1D15, rather than its total abundance, serves as the critical switch for mitochondria–lysosome homeostasis. CCCP induced a time-dependent biphasic change in TBC1D15 that persisted upon PINK1 knockdown, indicating regulation is not strictly PINK1-dependent. High-dose AuNPs trapped TBC1D15 on mitochondria and blocked lysosomal delivery via a PINK1-independent route. AuNPs exhibited biphasic concentration-dependent effects: low concentrations moderately upregulated TBC1D15, promoting adaptive organelle contacts, while millimolar concentrations caused pathological tethering. Subcellular fractionation and CQ intervention confirmed that defective lysosomal delivery underlies mitochondrial TBC1D15 accumulation upon AuNP challenge. Conclusions: This work establishes two distinct regulatory axes converging on TBC1D15. High-dose AuNPs can serve as a specific pharmacological blocker of TBC1D15 trafficking, offering therapeutic avenues for disorders characterized by impaired mitophagy.

## 1. Introduction

Mitochondrial quality control relies on dynamic crosstalk between mitochondria and lysosomes, and defects in this organelle communication drive mitochondrial dysfunction, neurodegeneration and metabolic disorders [1,2,3]. Mitophagy, the selective autophagic clearance of damaged mitochondria, acts as the core surveillance pathway to sustain organelle homeostasis, requiring coordinated mitochondrial fission and lysosomal degradation machinery [3,4]. Mitochondria–lysosome contact sites serve as physical platforms to mediate metabolite exchange, calcium signaling and autophagosome biogenesis, yet the molecular switches governing the formation and disassembly of these contacts remain incompletely characterized [5,6,7]. In particular, it remains unresolved whether the dynamic assembly and disassembly of these contact sites is primarily determined by the total cellular abundance of contact-site regulators or by their reversible subcellular trafficking between the two organelles.

TBC1 domain family member 15 (TBC1D15), a Rab7-specific GTPase-activating protein (GAP), is a pivotal dual regulator linking mitochondrial dynamics and lysosomal degradation [8,9,10,11]. As a Rab7-specific GAP, TBC1D15 directly controls mitochondria–lysosome contact disassembly by catalyzing Rab7 GTP hydrolysis, making it uniquely positioned to address this unresolved question. Accumulating evidence confirms TBC1D15 participates in mitochondrial fission and autophagosome–lysosome fusion by interacting with Fis1 and Rab7 [12,13]. Aberrant TBC1D15 expression disrupts organelle tethering and accelerates mitochondrial fragmentation; however, a critical unresolved contradiction remains: whether total cellular TBC1D15 protein abundance or its reversible subcellular shuttling between mitochondria and lysosomes functions as the primary determinant of mitochondrial homeostasis [14,15]. Existing studies only assess whole-cell TBC1D15 expression without separating organelle-specific protein pools, which cannot distinguish transcriptional/translational regulation from inter-organelle trafficking events. In addition, it remains unclear whether stressors trigger mitochondrial damage through shared or divergent TBC1D15-dependent cascades.

Gold nanoparticles (AuNPs) have emerged as versatile nanoscale tools to perturb mitochondria–lysosome communication, as they readily accumulate within cytoplasmic organelles and disrupt lysosomal hydrolase activity and mitochondrial redox balance [16,17,18]. Multiple reports have validated AuNP-induced organelle damage accompanied by altered TBC1D15 levels, yet no comparative framework exists to dissect whether AuNP toxicity converges on the canonical PTEN-induced kinase 1 (PINK1) mitophagy pathway or operates via an independent trafficking block [18]. The mitochondrial uncoupler CCCP represents the gold-standard inducer of PINK1-dependent mitophagy, which provides an ideal positive control to benchmark and contrasts AuNP-mediated mitochondrial injury [19,20]. Direct parallel comparison between CCCP and gradient-concentration AuNPs can resolve two key unanswered questions: (1) whether PINK1 signaling is mandatory for TBC1D15 remodeling under organelle stress; (2) whether AuNPs modify TBC1D15 abundance or block its mitochondria-to-lysosome translocation.

Beyond single-stressor treatments, autophagy blockade with chloroquine (CQ) and genetic silencing of PINK1 offer complementary approaches to dissect TBC1D15 turnover and subcellular distribution. Separate biochemical extraction of mitochondrial and lysosomal protein fractions enables direct quantification of compartmentalized TBC1D15 pools, overcoming the limitation of whole-cell lysis assays that obscure organelle-specific protein trafficking phenotypes [21]. In this work, we constructed a dual-stimulation experimental system using CCCP and serially diluted AuNPs in neuronal PC-12 and epithelial HeLa cell lines, combined with PINK1 siRNA knockdown, CQ autophagy inhibition and organelle subcellular fractionation. We aimed to systematically differentiate two distinct regulatory modes of TBC1D15 and define the core functional driver of mitochondria–lysosome crosstalk. This study clarifies that inter-organelle translocation, rather than global TBC1D15 expression, governs mitochondrial quality control, and establishes high-dose AuNPs as a unique PINK1-independent inhibitor of TBC1D15 trafficking, providing new mechanistic insights into nanoparticle-triggered organelle pathology [22,23] and candidate therapeutic strategies for PINK1-deficient mitochondrial disorders [24,25,26].

## 2. Materials and Methods

### 2.1. Preparation and Characterization of AuNPs

Gold nanoparticles (AuNPs) were synthesized following our previously reported procedure with slight modifications [18]. In a typical synthesis, 100 mL of ultrapure water was heated to boiling in a beaker, after which 1 mL of 1% (*w*/*v*) HAuCl_4_ solution (Shanghai Macklin Biochemical Co., Ltd., Shanghai, China) was swiftly introduced under vigorous stirring. Subsequently, 3 mL of 1% (*w*/*v*) trisodium citrate solution (Shandong Xiya Chemical Industry Co., Ltd., Linyi, China) was added, and the mixture was maintained at boiling temperature. The solution gradually transitioned from pale yellow to a greyish-blue and eventually to a distinct cherry-red hue upon continued refluxing. After 30 min of boiling, 5 mL of 0.1% (*w*/*v*) ascorbic acid solution (Shanghai Aladdin Biochemical Technology Co., Ltd., Shanghai, China) was added, and the reaction was allowed to proceed for an additional 1 min with stirring to complete the synthesis. To eliminate residual reducing agents, the as-prepared AuNPs were subjected to centrifugation at 20,000 rpm for 10 min prior to cell-based experiments.

The obtained AuNPs were thoroughly characterized using multiple analytical techniques. Morphology and particle size distribution were examined via transmission electron microscopy (TEM, JEM-F200, JEOL Ltd., Akishima, Tokyo, Japan). The optical absorption properties were evaluated by UV–visible spectrophotometry (UV-5500PC, Shanghai Metash Instruments Co., Ltd., Shanghai, China). Surface charge (ζ-potential) and hydrodynamic diameter in ultrapure water were determined using a Zetasizer Nano Z and a Zetasizer Nano S90 (Malvern Panalytical Ltd., Malvern, UK), respectively. All AuNP concentrations used in this study are expressed as elemental gold concentration (nM, μM, and mM), unless otherwise stated.

### 2.2. Cell Line and RNA Interference

The neural cell model employed in this study was the PC-12 (High differentiation) (rat adrenal pheochromocytoma cell line highly differentiated) (Catalog No. CL-0481) purchased from Wuhan Procell Life Science & Technology Co., Ltd., Wuhan, China. HeLa cells (human cervical adenocarcinoma line) (Catalog No. STCC10603P) purchased from Wuhan Servicebio Technology Co., Ltd., Wuhan, China, were utilized as a non-neuronal control cell line in this study.

Cells were seeded in 6-well cell culture plates and transfected with PINK1 siRNA when they reached approximately 70% confluence. The PINK1-targeting siRNAs used were as follows: for PC-12 cells, PINK1 #1 (5′-UUUGCUGAACCCAAGGCUU-3′), PINK1 #2 (5′-GGUGUUUGGCCUUCUCCAG-3′), PINK1 #3 (5′-GCAAUUUUUACACAGAAAA-3′). For HeLa cell line, PINK1 #1 (5′-CCGCAAAUGUGCUUCAUCU-3′), HeLa PINK1 #2 (5′-CCUCGUUAUGAAGAACUAU-3′), and HeLa PINK1 #3 (5′-GUCACUUACAGAAAAUCCA-3′). For each cell line, scrambled siRNAs were used as a negative control as follows: PC-12 scrambled siRNA (5′-CUACGUCAUUGGCAAGCU-3′), and HeLa scrambled siRNA (5′-GCUACGUACUGCAAUCGCU-3′). The scrambled sequences were designed to have the same nucleotide composition as the corresponding PINK1 siRNAs but with no significant homology to any known gene in the respective species (rat for PC-12; human for HeLa). All siRNAs were purchased from Sangon Biotech Co., Ltd., Shanghai, China. Each siRNA was dissolved in DEPC water (Sangon Biotech (Shanghai) Co., Ltd., Shanghai, China) to prepare a 10 μM stock solution for later use. Lipofectamine RNAiMAX reagent (Thermo Fisher Scientific, Waltham, MA, USA) was diluted in Opti-MEM medium (Thermo Fisher Scientific, Waltham, MA, USA) in a 1.5 mL sterile nuclease-free centrifuge tube (Axygen, Union City, CA, USA). Another aliquot of Opti-MEM medium was dispensed into a 1.5 mL sterile nuclease-free centrifuge tube, and the siRNA stock solution was added. The diluted Lipofectamine RNAiMAX reagent was then mixed with the siRNA dilution at a 1:1 ratio. The original cell culture medium was discarded, and the cells were washed twice with PBS (Beijing Solarbio Science & Technology Co., Ltd., Beijing, China). Subsequently, 700 μL of serum-free and antibiotic-free basal medium was added, followed by the addition of 300 μL of the mixture of siRNA and Lipofectamine RNAiMAX reagent dilution, which had been incubated for 5 min. The cells were then placed in an incubator (Form 310; Eppendorf AG, Hamburg, Germany) for 6 h, after which the medium was replaced with complete medium containing serum. The scrambled siRNA was used at the same final concentration as the PINK1 siRNAs. The siRNA used in this study, including their suppliers, catalogue numbers, and sequences, are listed in Supplementary Materials (Table S1).

### 2.3. RIPA-Soluble Extracts Protocol

Ice, pre-labeled 1.5 mL centrifuge tubes, and cell scrapers were prepared before the experiment. RIPA lysis buffer (Beijing Solarbio Science & Technology Co., Ltd., Beijing, China) (1 mL) was supplemented with PMSF (Beijing Solarbio Science & Technology Co., Ltd., Beijing, China) (10 μL, 1 mM final concentration). Cultured cell plates were removed from the incubator and washed twice with ice-cold PBS, ensuring complete PBS aspiration. Ice-cold lysis buffer (200 μL/well) was added to each well, and the plates were incubated on ice. Cells were mechanically detached using cell scrapers and thoroughly homogenized by pipetting. The lysate was transferred to prepared centrifuge tubes and maintained on ice with intermittent agitation. Brief ultrasonication (10–15 s, 20% amplitude, 0.5 s pulse interval) was performed to ensure complete membrane disruption. After 25 min of ice incubation, samples were centrifuged at 14,000× *g* (4 °C) for 4 min. The supernatant was carefully transferred to fresh tubes and mixed with 4× Laemmli buffer (50 μL buffer per 150 μL lysate) by vigorous pipetting. Proteins were denatured by heating at 100 °C for 10 min using a dry bath incubator. Processed samples were stored at −20 °C until further analysis.

### 2.4. Mitochondrial Protein Extraction

The same batch of treated cells was divided into two aliquots for mitochondrial and lysosomal extraction, respectively. Mitochondrial protein extraction was performed using a commercial isolation kit (Proteintech Group, Inc., Wuhan, China). Cells were seeded in 15 cm culture dishes and cultured overnight at 37 °C under 5% CO_2_. The original medium was replaced with 15 mL fresh complete medium or media containing AuNPs (1 mM), CCCP (MedChemExpress, Monmouth Junction, NJ, USA) (20 μM), or CQ (25 μM), followed by incubation for specified durations (3, 12, 24 h). Mitochondrial isolation buffer components were thawed to room temperature and subsequently chilled on ice, with centrifuges pre-cooled to 4 °C. Cells were mechanically detached using a cell scraper, collected by centrifugation at 500× *g* (4 °C, 5 min), and washed twice with ice-cold PBS. The cell pellet was resuspended in 1 mL ice-cold Mitochondrial Isolation Reagent A and homogenized using a pre-chilled glass homogenizer with 10 strokes under ice-cold conditions. The homogenate was layered over 0.9 mL Mitochondrial Isolation Reagent B and centrifuged at 600× *g* (4 °C, 10 min). The supernatant was transferred to new tubes and centrifuged at 10,000× *g* (4 °C, 10 min) to pellet mitochondria. The mitochondrial fraction was lysed in 100 μL ice-cold mitochondrial lysis buffer for 30 min on ice. Lysates were mixed with SDS-PAGE loading buffer (3:1 ratio), denatured at 100 °C for 5 min, and either used immediately or stored at −80 °C.

### 2.5. Lysosomal Protein Extraction

The lysosomal protein extraction procedure referenced the cellular drug administration method outlined in Section 2.4. Cells were detached using a cell scraper, collected via centrifugation at 500× *g* (4 °C, 5 min), and washed twice with ice-cold PBS. The pellet was resuspended in 1 mL ice-cold Reagent A (Lysosomal Protein Extraction Kit; Beijing Solarbio Science & Technology Co., Ltd., Beijing, China) and incubated on ice with continuous agitation for 10 min. Homogenization was performed using a pre-chilled glass homogenizer with approximately 30 strokes under ice-cold conditions. The homogenate was centrifuged at 1000× *g* (4 °C, 5 min), and the supernatant was retained while discarding the pellet. Subsequent centrifugation steps included: (1) 4000× *g* (4 °C, 10 min) to remove residual debris, (2) 13,400× *g* (4 °C, 25 min) to pellet the crude lysosomal fraction, followed by resuspension in 500 μL ice-cold Reagent B and centrifugation at 13,400× *g* (4 °C, 20 min). The resulting pellet was solubilized in 100 μL Reagent C with gentle vortexing for 25 min at 4 °C, then centrifuged at 12,000× *g* (4 °C, 15 min). The purified lysosomal protein supernatant was combined with SDS-PAGE loading buffer, denatured at 100 °C for 10 min using a heating block, and stored at −80 °C for downstream applications.

### 2.6. Western Blot Analysis

SDS-PAGE gels with 10 wells were prepared for protein separation. Each lane was loaded with 20 μL of RIPA-soluble protein extract and 5 μL of pre-stained molecular weight marker (ABclonal Technology Co., Ltd., Wuhan, China). Electrophoresis was initially performed at 80 V for 30 min, followed by 130 V for 1.5 h. Proteins were transferred to nitrocellulose membranes (Beijing Solarbio Science & Technology Co., Ltd., Beijing, China) using a semi-dry transfer system at 220 mA for 1.5 h. Post-transfer, membranes were blocked with 25 mL of blocking buffer (5% non-fat dry milk in TBST) at room temperature for 1 h. Three 5 min washes with 15 mL TBST (10× TBST buffer; Wuhan Servicebio Technology Co., Ltd., Wuhan, China) were performed before incubating membranes with primary antibody solution (10 mL, diluted in TBST containing 1% BSA) overnight at 4 °C with gentle agitation. Membranes were subsequently washed three times with TBST (15 mL, 5 min/wash) and incubated with secondary antibody (1:10,000 dilution in TBST) under light-protected conditions at room temperature for 1 h. Following final TBST washes, protein bands were visualized using an Odyssey DLx Infrared Imaging System (LI-COR Biosciences, Lincoln, NE, USA). The primary and secondary antibodies used in this study, including their suppliers and catalogue numbers, are listed in Supplementary Materials (Table S1).

### 2.7. Quantification and Statistical Analysis

All the Western blot images were quantified using the Image Studio software (version 5.2; LI-COR Biosciences, Lincoln, NE, USA) that comes with the scanning instrument’s infrared laser imaging system (Odyssey DLX; LI-COR Biosciences, Lincoln, NE, USA). The grayscale values of the Western blot bands were automatically read. Background subtraction was applied using Image Studio default settings, and band intensities were normalized to loading controls. The linearity of the signal was confirmed by serial dilution of representative samples. Data were analyzed using Multiple *t*-tests in GraphPad Prism 8.0.2, with correction for multiple comparisons using the Holm-Šídák method. Data were presented as the mean ± standard deviation (SD). Statistical significance was determined at *p* ≤ 0.05. All experiments were performed with at least three independent biological replicates (*n* = 3).

## 3. Results

### 3.1. Autophagy Modulates the Stability and Turnover of Cellular TBC1D15

In our previous work, AuNPs were shown to induce autophagy, as evidenced by a marked increase in LC3-II levels, and were also associated with notable alterations in TBC1D15 expression (independent validation; details are provided in Appendix A) [18]. In the present study, we first investigated whether autophagy modulation affects TBC1D15 expression, using CCCP and chloroquine (CQ) in PC-12 and HeLa cells.

**Figure 1:**
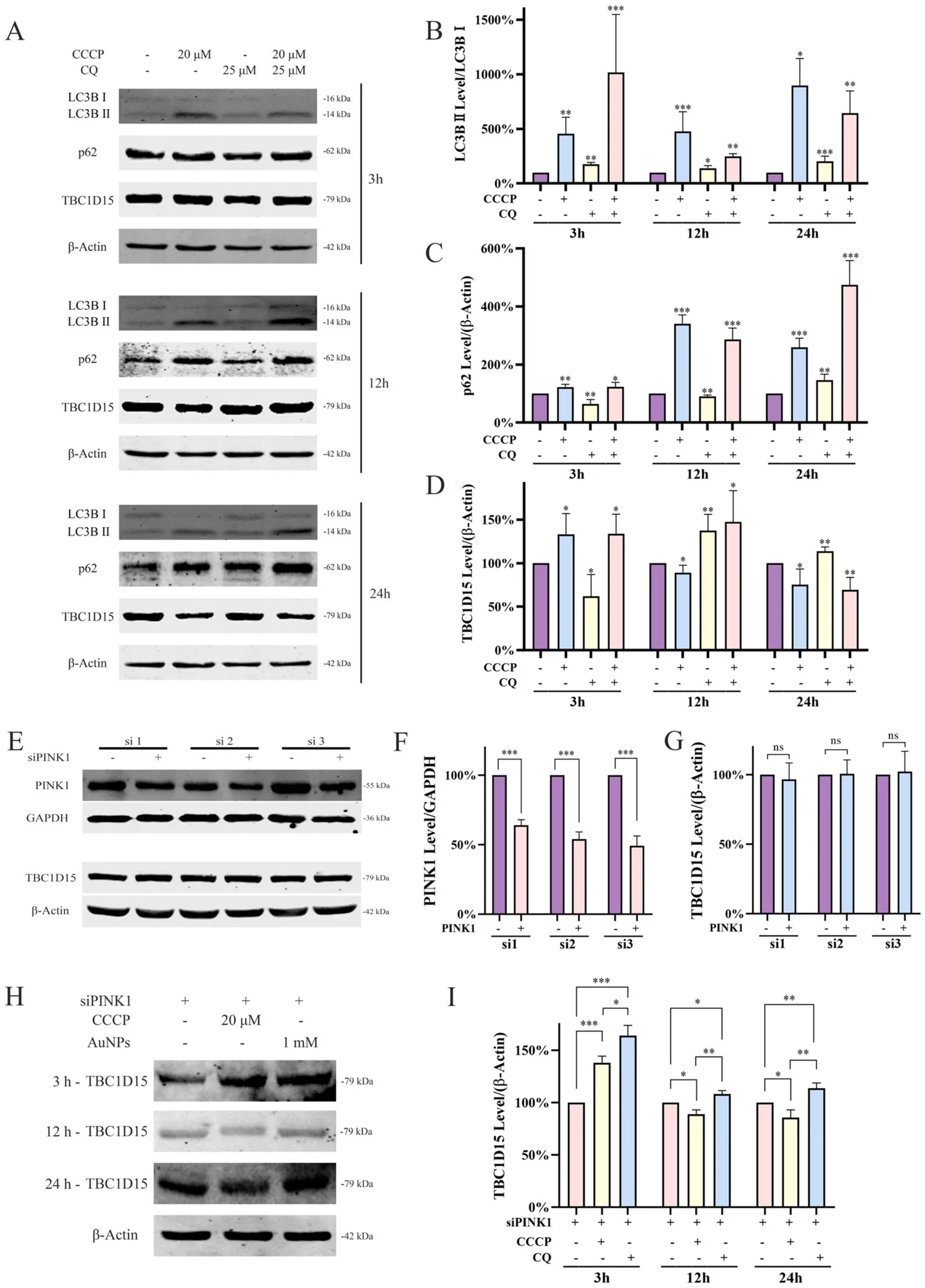
Western blot analysis of autophagy markers and TBC1D15 in PC−12 cells. (**A**) Representative immunoblots of LC3B−II, p62, TBC1D15, and β−Actin in PC−12 cells treated with CCCP (20 μM), CQ (25 μM), or CCCP+CQ for 3, 12, and 24 h. (**B**–**D**) Densitometric quantification of (**B**) LC3B−II/LC3B−I ratios, (**C**) p62/β−Actin, and (D) TBC1D15/β−Actin ratios at the indicated time points. (**E**) Immunoblots showing PINK1 and TBC1D15 expression in PC−12 cells 72 h after transfection with three distinct siPINK1 constructs (si1, si2, si3) or scrambled siRNA control. GAPDH and β−Actin were used as loading controls for PINK1 and TBC1D15, respectively. (**F**,**G**) Densitometric quantification of (**F**) PINK1/GAPDH and (**G**) TBC1D15/β−Actin ratios. (**H**) Immunoblots of TBC1D15 and β−Actin in siPINK1−transfected PC−12 cells subsequently treated with CCCP (20 μM) or AuNPs (1 mM) for 3, 12, and 24 h. (**I**) Densitometric quantification of TBC1D15/β−Actin ratios corresponding to (H). Data are presented as mean ± SD (*n* = 3). Statistical significance: ns, not significant (*p* > 0.05); * *p* ≤ 0.05; ** *p* ≤ 0.01; *** *p* ≤ 0.001. LC3B−II/β−Actin and LC3B−I/β−Actin quantifications are provided in Supplementary Figures S1 and S2.

In PC-12 cells, all treatments consistently elevated LC3B-II/LC3B-I ratios across all time points examined (Figure 1B). At 3 h, CCCP increased LC3B-II/LC3B-I ratio, p62, and TBC1D15; both CCCP and CCCP+CQ upregulated these three proteins, whereas CQ alone decreased p62 and TBC1D15 (Figure 1A–D). At 12 h, p62 remained elevated under CCCP or CCCP+CQ, while TBC1D15 showed opposing responses—decreasing with CCCP but increasing with CQ (Figure 1A–D). At 24 h, CQ alone raised p62, whereas CCCP+CQ reduced TBC1D15 (Figure 1A–D). Although LC3B-II/LC3B-I ratio remained persistently elevated throughout, both p62 and TBC1D15 exhibited time-dependent fluctuations, suggesting differential regulation of autophagy markers and TBC1D15 over time.

**Figure 2:**
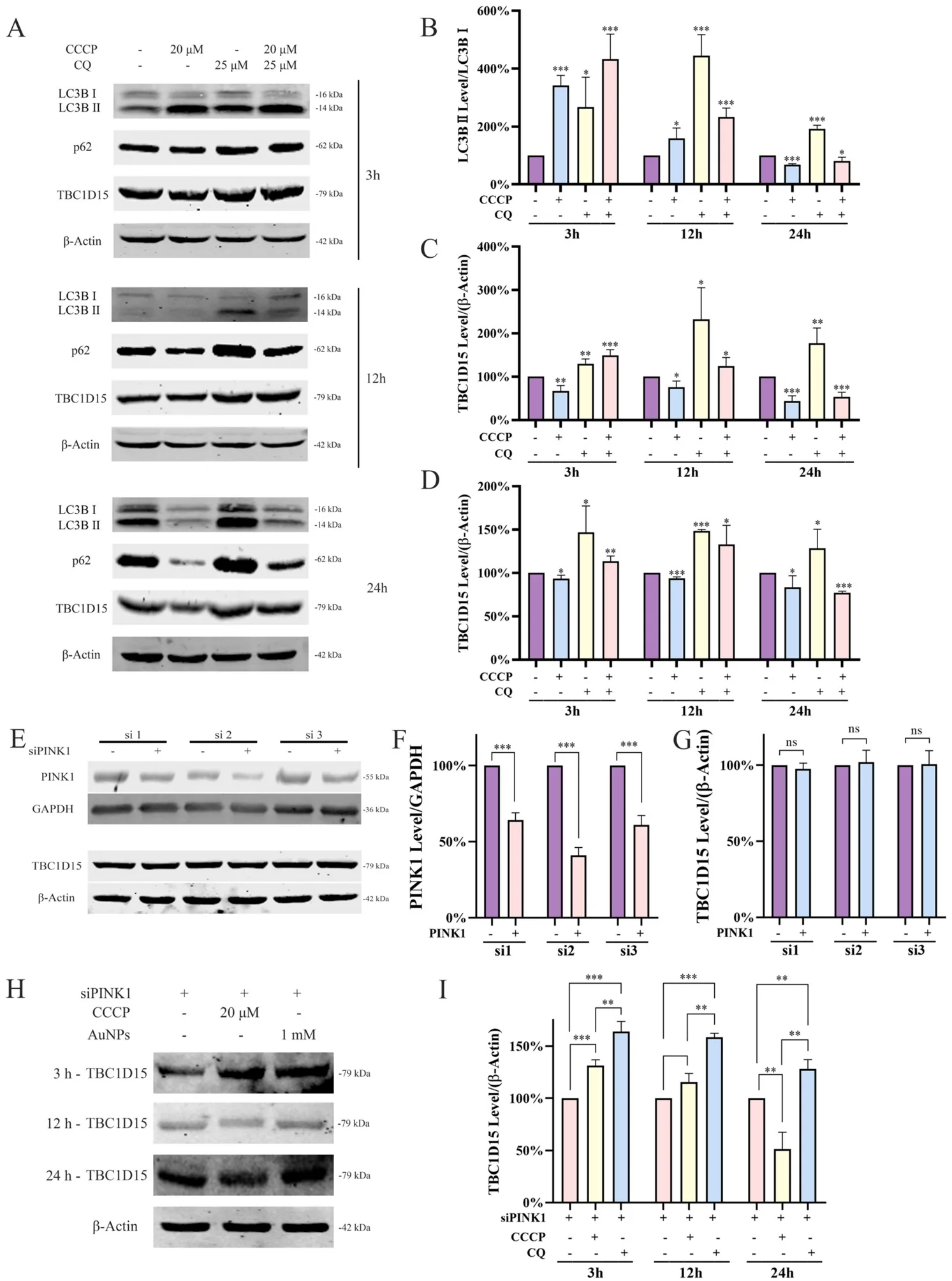
Western blot analysis of autophagy markers and TBC1D15 in HeLa cells. (**A**) Representative immunoblots of LC3B−II, p62, TBC1D15, and β−Actin in HeLa cells treated with CCCP (20 μM), CQ (25 μM), or CCCP+CQ for 3, 12, and 24 h. (**B**–**D**) Densitometric quantification of (**B**) LC3B−II/LC3B−I ratio, (**C**) p62/β−Actin, and (**D**) TBC1D15/β−Actin ratios at the indicated time points. (**E**) Immunoblots showing PINK1 and TBC1D15 expression in HeLa cells 72 h after transfection with three distinct siPINK1 constructs (si1, si2, si3) or scrambled siRNA control. GAPDH and β−Actin were used as loading controls for PINK1 and TBC1D15, respectively. (**F**,**G**) Densitometric quantification of (**F**) PINK1/GAPDH and (**G**) TBC1D15/β−Actin ratios. (**H**) Immunoblots of TBC1D15 and β−Actin in siPINK1−transfected HeLa cells subsequently treated with CCCP (20 μM) or AuNPs (1 mM) for 3, 12, and 24 h. (**I**) Densitometric quantification of TBC1D15/β−Actin ratios corresponding to (H). Data are presented as mean ± SD (*n* = 3). Statistical significance: ns, not significant (*p* > 0.05); * *p* ≤ 0.05; ** *p* ≤ 0.01; *** *p* ≤ 0.001. LC3B−II/β−Actin and LC3B−I/β−Actin quantifications are provided in Supplementary Figures S3 and S4.

In HeLa cells, LC3B-II/LC3B-I ratio was upregulated at 3 and 12 h but decreased after 24 h with CCCP or CCCP+CQ, remaining elevated with CQ alone (Figure 2B). At 3 h, CCCP reduced p62 and TBC1D15, while CQ (alone or combined) increased them (Figure 2A–D). This pattern persisted at 12 h, with CQ enhancing p62 accumulation (Figure 2A–D). By 24 h, CCCP+CQ reduced both p62 and TBC1D15, while other treatments maintained prior expression levels (Figure 2A–D).

### 3.2. PINK1 Knockdown Does Not Regulate Basal TBC1D15 Abundance and Fails to Reverse AuNPs Mediated TBC1D15 Retention

The PINK1-mediated pathway is a central regulator of mitochondrial autophagy. To investigate whether TBC1D15 functions downstream of or in parallel to this pathway, we first validated the efficiency of siRNA-mediated PINK1 knockdown and then examined its effect on TBC1D15 protein levels (Figure 1E–G and Figure 2E–G). In both PC-12 and HeLa cells, all three siPINK1 constructs effectively suppressed PINK1 expression relative to scrambled siRNA control (Figure 1E,F and Figure 2E,F). However, TBC1D15 levels remained unchanged following PINK1 depletion in both cell lines (Figure 1G and Figure 2G), indicating that TBC1D15 expression is not directly regulated by PINK1 under these experimental conditions.

We then examined TBC1D15 expression in siPINK1-transfected cells treated with CCCP or AuNPs. In PC-12 cells, TBC1D15 increased within 3 h, with AuNPs sustaining higher expression than CCCP over 24 h (Figure 1H,I). By 12–24 h, CCCP reduced TBC1D15 relative to siPINK1-transfected control and AuNPs-treated cells (Figure 1H,I). In HeLa cells, both AuNPs and CCCP elevated TBC1D15 at 3–12 h (Figure 2H,I). After 24 h, CCCP reduced TBC1D15 below baseline, whereas AuNPs maintained elevated expression (Figure 2H,I).

### 3.3. Subcellular Distribution Shifts in TBC1D15 in Mitochondrial and Lysosomal Fractions

We analyzed TBC1D15 distribution in mitochondrial and lysosomal fractions from PC-12 cells treated with AuNPs, CCCP, CQ, or their combinations.

**Figure 3:**
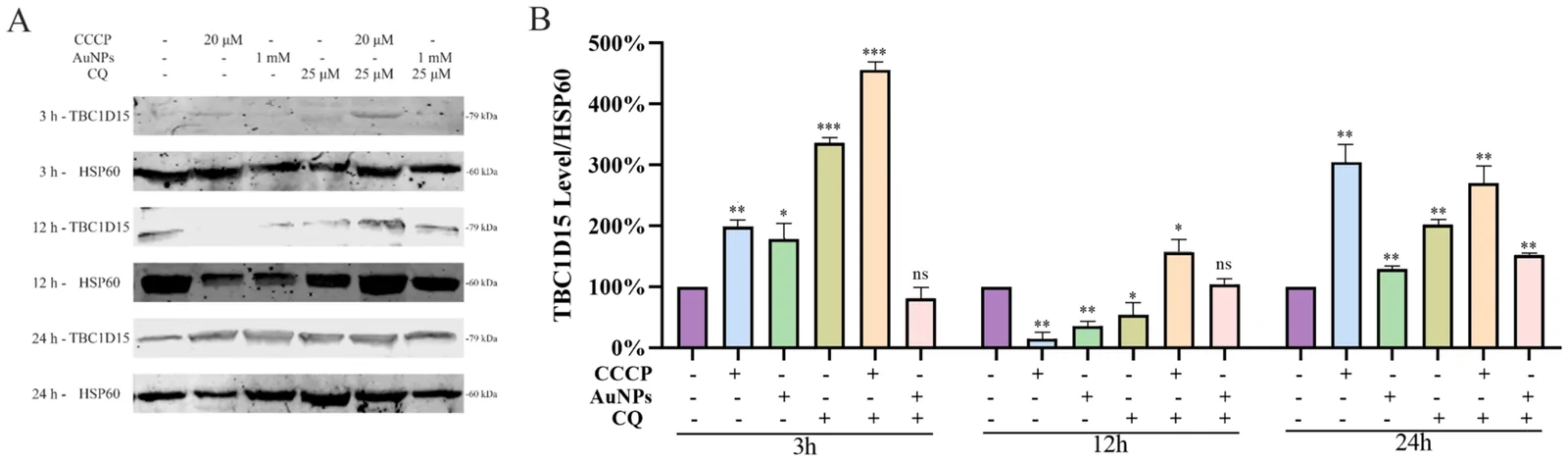
Western blot analysis of TBC1D15 and HSP60 in PC−12 cells. (**A**) Representative immunoblots of mitochondrial TBC1D15 and HSP60 in PC−12 cells treated with CCCP (20 μM), AuNPs (1 mM), CQ (25 μM), CCCP+CQ, or AuNPs+CQ for 3, 12, and 24 h. (**B**) Densitometric quantification of mitochondrial TBC1D15/HSP60 ratios at the indicated time points. Data are presented as mean ± SD (*n* = 3). Statistical significance: ns, not significant (*p* > 0.05); * *p* ≤ 0.05; ** *p* ≤ 0.01; *** *p* ≤ 0.001.

In mitochondrial fractions, TBC1D15 signals were detectable at all time points, with prominent changes observed at 3 and 24 h (Figure 3A). Densitometric quantification of mitochondrial TBC1D15 normalized to 60 kDa heat shock protein (HSP60) revealed that at 3 h, CCCP or AuNPs treatment significantly increased mitochondrial TBC1D15 compared to control (Figure 3B). CQ alone or in combination with CCCP also elevated mitochondrial TBC1D15, whereas AuNPs+CQ did not significantly increase it. At 12 h, mitochondrial TBC1D15 decreased under CCCP, AuNPs, or CQ alone, but remained elevated under CCCP+CQ; AuNPs+CQ showed no significant change. After 24 h, all treatments increased mitochondrial TBC1D15 above control levels (Figure 3B).

**Figure 4:**
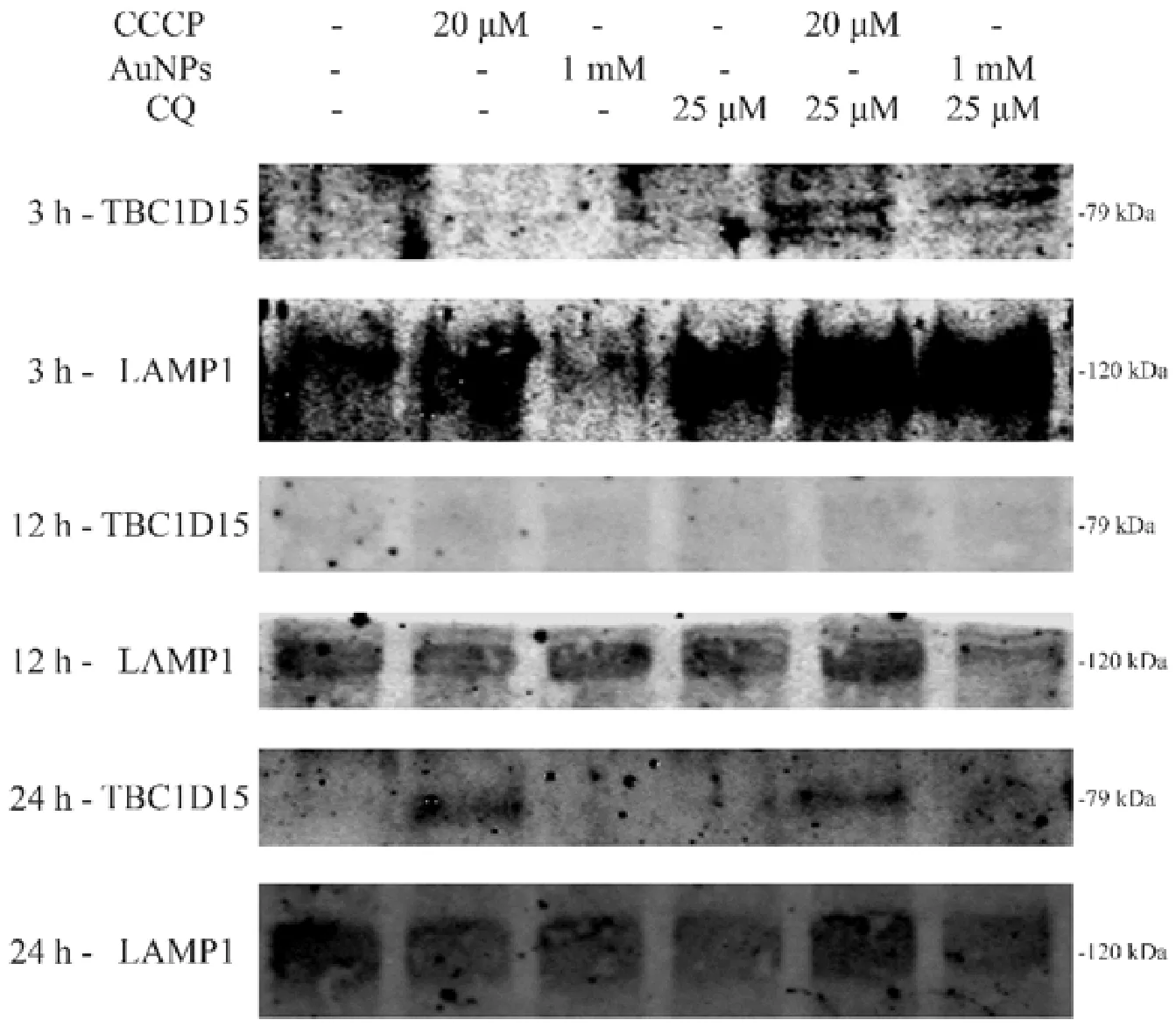
Western blot analysis of TBC1D15 and LAMP1 in PC−12 cells. Representative immunoblots of lysosomal TBC1D15 and LAMP1 in PC−12 cells treated with CCCP (20 μM), AuNPs (1 mM), CQ (25 μM), CCCP+CQ, or AuNPs+CQ for 3, 12, and 24 h.

In lysosomal fractions, TBC1D15 was undetectable in most single-treatment groups, consistent with rapid lysosomal degradation. However, when lysosomal degradation was blocked by CQ, TBC1D15 became detectable (Figure 4): at 3 h, clear bands were observed in the CCCP+CQ and AuNPs+CQ groups; at 12 h, TBC1D15 remained undetectable in all groups, consistent with the decline in total TBC1D15 at this time point (Figure 1A,D); at 24 h, clear bands were observed in the CCCP and CCCP+CQ groups, with a faint band in the AuNPs+CQ group. These qualitative observations suggest that TBC1D15 traffics to lysosomes and is subject to lysosomal degradation, and that AuNPs partially impair this trafficking. Due to the low signal intensity and impurity of LAMP1 bands, densitometric quantification was not performed; these data are therefore interpreted qualitatively. Because functional TBC1D15 rapidly translocates into lysosomes for autophagic degradation upon mitochondria–lysosome tethering, its steady-state lysosomal abundance is extremely low. Under AuNP treatment, TBC1D15 is retained on mitochondria, and its lysosomal delivery is blocked, which is consistent with the minimal lysosomal TBC1D15 signal observed. However, due to the limited detectability, these data should be interpreted as supportive rather than definitive evidence of reduced lysosomal delivery.

## 4. Discussion

### 4.1. Distinct Regulatory Modes of TBC1D15 Modulated by CCCP and High Concentration AuNPs

The present work identifies TBC1D15 as an important molecular regulator of mitochondria–lysosome communication and mitochondrial quality control [27,28,29]. By combining pharmacological and genetic interventions, we distinguish two regulatory routes that modulate TBC1D15 expression and localization, highlighting its role in autophagy-dependent mitochondrial homeostasis. Rather than acting as a sole “central coordinator,” our data support a model in which TBC1D15 subcellular trafficking serves as a critical determinant of organelle crosstalk, while additional factors likely contribute to the overall regulation.

In response to the mitochondrial uncoupler CCCP, immunoblot analysis revealed cell-type-specific and context-dependent changes in TBC1D15 levels. In PC-12 cells under non-knockdown conditions, CCCP increased TBC1D15 at 3 h, followed by a decline at 12 and 24 h. In contrast, in HeLa cells under the same conditions, CCCP reduced TBC1D15 at 3 h. Notably, upon siRNA-mediated PINK1 knockdown, both PC-12 and HeLa cells exhibited a biphasic response to CCCP, with a sharp increase at 3 h and a progressive decline at 12 and 24 h (Figure 1H,I and Figure 2H,I). This biphasic response persisted even after PINK1 knockdown, with TBC1D15 remaining significantly elevated over scrambled siRNA controls at 3 h and significantly reduced at 12 and 24 h. These results suggest that CCCP-induced TBC1D15 regulation is not strictly dependent on PINK1; while the canonical PINK1/Parkin mitophagy axis may participate, additional PINK1-independent mechanisms also contribute to the observed fluctuations. It should be noted, however, that the PINK1 knockdown efficiency, although significant, was incomplete (Figure 1E,F and Figure 2E,F), and residual PINK1 may still partially influence the response. Therefore, the term “PINK1-independent” should be interpreted with caution, and further validation using PINK1-knockout models (e.g., MEFs) would strengthen this conclusion. This finding nonetheless revises the conventional view that CCCP acts solely through PINK1, and points to a more complex regulatory network governing TBC1D15 turnover under mitochondrial stress.

In contrast, we employed AuNPs as a tool to perturb TBC1D15 function, building on our previous work which established that AuNPs disrupt mitochondrial homeostasis by interfering with TBC1D15 [18]. In the current study, subcellular fractionation data showed that high-concentration AuNP treatment increased TBC1D15 in the mitochondrial fraction, whereas lysosomal TBC1D15 was largely undetectable in AuNP-treated cells without CQ and became detectable only upon CQ co-treatment (Figure 3 and Figure 4). These observations are consistent with the interpretation that TBC1D15 is retained on mitochondria and its lysosomal delivery is impaired. However, because lysosomal TBC1D15 signals were generally low or undetectable in cells treated without CQ—including untreated controls—we cannot definitively conclude that AuNPs “further reduce” lysosomal TBC1D15 below this baseline. Instead, the data suggest that under these conditions, TBC1D15 is predominantly associated with mitochondria, and its translocation to lysosomes is limited. Additional controls, such as lysosomal enrichment markers (lysosomal-associated membrane protein 1, LAMP1) and a positive control for lysosomal TBC1D15 detection, will be necessary in future studies to confirm this interpretation. Importantly, PINK1 knockdown did not reverse any of these AuNP-induced effects, supporting the notion that the AuNP-mediated disruption operates largely through a PINK1-independent route. However, possible off-target effects of AuNPs, such as lysosomal destabilization or membrane perturbation, cannot be excluded at this stage. Collectively, our findings position TBC1D15 as a key effector of mitochondria-linked autophagy, and unveil a noncanonical pathway that may complement existing disease models of impaired mitochondria-lysosome communication in neurodegenerative disorders [30,31].

Notably, the temporal patterns of LC3B-II/LC3B-I ratio, p62, and TBC1D15 differed between PC-12 and HeLa cells. In PC-12 cells, p62 continued to accumulate under combined CCCP+CQ treatment over time, and the increase was greater than with either treatment alone (Figure 1A–D). This likely reflects a synergistic effect: CCCP induces autophagosome formation, while CQ blocks autophagic flux, leading to enhanced accumulation of autophagic substrates. In contrast, HeLa cells showed a less pronounced p62 accumulation and a more rapid decline in LC3B-II/LC3B-I ratio at 24 h (Figure 2A–D), suggesting cell-type-specific differences in autophagic capacity or mitochondrial stress responses. These differential responses may also reflect distinct baseline autophagic flux or lysosomal degradation efficiency between neuronal-like PC-12 cells and epithelial HeLa cells.

These findings are conceptually related to recent work from Qian Cai’s group, which showed that reduced TBC1D15 abundance in tauopathy neurons causes hyper-tethering of autophagosome–mitochondria contacts and impairs autophagosome transport, leading to p62 accumulation [32]. While that study emphasizes TBC1D15 abundance and autophagosome–mitochondria contacts, our data highlight a distinct regulatory layer: the subcellular trafficking of TBC1D15 between mitochondria and lysosomes. Under high-dose AuNP treatment, total TBC1D15 levels remained unchanged, yet mitochondrial retention and impaired lysosomal delivery were evident. Together, these findings suggest that TBC1D15 dysfunction can arise from either altered abundance or blocked trafficking, both converging on impaired autophagic clearance.

### 4.2. Concentration-Dependent Dual Effects of AuNPs on TBC1D15-Mediated Mitochondrial Homeostasis

Our time- and concentration-gradient experiments suggest a biphasic biological response of TBC1D15 to AuNP treatment, which may help reconcile the seemingly contradictory outcomes of low- versus high-dose nanoparticle exposure reported in current nanotoxicology studies [33]. At a low concentration (5 nM), AuNPs moderately elevate TBC1D15 protein levels, which we hypothesize may promote adaptive mitochondria–lysosome tethering and enhance basal mitophagy. This hypothesis is supported by our previous findings that 5 nM AuNPs increased mitochondria–lysosome colocalization, lysosome number, and TBC1D15 expression in PC-12 cells [18]. However, direct tethering or mitophagy measurements under this low-dose condition were not repeated in the present study; therefore, this interpretation remains a hypothesis to be tested in future work using live-cell imaging or proximity ligation assays.

In contrast, millimolar concentrations of AuNPs were associated with pronounced accumulation of TBC1D15 in the mitochondrial fraction and minimal lysosomal signal, suggesting a pathological over-stabilization of mitochondria–lysosome contact sites. This persistent tethering may trap TBC1D15 on the mitochondrial outer membrane, preventing its normal detachment and subsequent lysosomal delivery for autophagic degradation. The accumulated TBC1D15 may disturb the mitochondrial fission–fusion balance, trigger excessive fragmentation, and aggravate organelle functional collapse. However, our current data do not provide direct structural evidence of “jammed” contact sites; further ultrastructural and quantitative dose–response studies are needed to validate this model. Importantly, because we have previously established that AuNPs directly interfere with TBC1D15 to disrupt mitochondrial homeostasis [18], here we exploit this concentration-switchable property of AuNPs as an experimental tool to block TBC1D15 trafficking. Nonetheless, the possibility that AuNPs exert additional TBC1D15-independent effects remains, and future work should address this by using TBC1D15 knockdown or knockout models.

### 4.3. Study Limitations and Future Research Perspectives

Although our cellular data indicate that the subcellular localization of TBC1D15–rather than its total cellular abundance–is an important determinant for maintaining mitochondrial homeostasis [27], several limitations warrant further investigation. First, the precise molecular interaction between AuNPs and TBC1D15 remains incompletely characterized. Future in vitro biochemical assays (e.g., surface plasmon resonance, SPR) and high-resolution laser scanning confocal microscopy will clarify whether AuNPs directly bind TBC1D15 or indirectly alter mitochondrial membrane properties to hinder its translocation. Second, all current experiments were restricted to immortalized cell lines (PC-12 and HeLa). Primary neuronal cultures and in vivo animal models are required to validate the physiological relevance of the TBC1D15-centric regulatory axis under pathological conditions, particularly because calcium-dependent organelle signaling pathways differ substantially between cell lines and primary tissues [34,35]. Third, although our autophagic flux assessment primarily relied on conventional LC3B-II and p62 immunoblotting, we did not perform tandem mRFP-GFP-LC3 fluorescence detection in this study. Instead, we quantified both LC3-I and LC3-II and calculated the LC3-II/LC3-I ratio, which directly reflects LC3 lipidation and autophagosome formation. This ratio is presented in the main figures, while LC3-I/β-Actin and LC3-II/β-Actin quantifications are provided in the Supplementary Figure S1. Future studies incorporating tandem mRFP-GFP-LC3 fluorescence detection and live-cell confocal imaging of mitochondria–lysosome colocalization will be necessary to dynamically monitor autophagic flux and organelle contact changes. Fourth, we did not include a transfection-reagent-only control or CQ treatment in the siPINK1 background. Although scrambled siRNA was used as the negative control and the transfection reagent amount was kept constant, these additional controls would further strengthen the interpretation of the siRNA experiments. Future studies incorporating these controls will help clarify the interplay between PINK1 signaling and autophagic flux in regulating TBC1D15 dynamics. Fifth, the subcellular fractionation experiments lacked purity controls (e.g., TOM20, LAMP1, GAPDH exclusion), and the lysosomal TBC1D15 signal was at the detection limit. Quantitative densitometric analysis of mitochondrial and lysosomal fractions, along with appropriate positive controls, will be essential to strengthen the trafficking conclusions. Finally, the low-dose AuNP-induced adaptive tethering hypothesis requires direct functional validation, such as colocalization imaging and mitophagy flux assays.

From a translational standpoint, the identification of high-dose AuNPs as a specific functional inhibitor of TBC1D15 translocation offers a promising strategy for treating mitochondrial disorders caused by deficiencies in the canonical PINK1/Parkin pathway. Targeted modulation of TBC1D15 subcellular trafficking is expected to bypass defective mitophagy signaling and restore impaired mitochondrial quality control in related metabolic and neurodegenerative diseases. However, given the potential off-target effects of AuNPs, alternative and more specific TBC1D15 inhibitors should be explored in future studies.

## 5. Conclusions

In this work, we delineate two regulatory modes converging on TBC1D15-mediated mitochondria–lysosome crosstalk, extending our previous study that focused on AuNP cytotoxicity [18]. Our data indicate that the subcellular trafficking of TBC1D15, rather than its total intracellular abundance, is an important determinant of mitochondrial quality control.

High-dose AuNPs increased TBC1D15 in the mitochondrial fraction, whereas lysosomal TBC1D15 was largely undetectable in AuNP-treated cells without CQ and was detected only upon CQ co-treatment, consistent with impaired lysosomal delivery. These effects were not reversed by PINK1 knockdown, supporting a PINK1-independent mechanism [36,37], although possible off-target effects of AuNPs cannot be excluded. Low-dose AuNPs moderately elevated TBC1D15 levels, which we hypothesize may promote adaptive organelle contacts; however, direct evidence for enhanced tethering or mitophagy under this condition is currently lacking. This biphasic concentration-dependent bioactivity [33,38] warrants further investigation.

Collectively, this study provides a comparative framework separating PINK1-dependent and PINK1-independent modes of TBC1D15 regulation. High-dose AuNPs may serve as an experimental tool to block TBC1D15 trafficking, but their lack of specificity should be considered. These findings position TBC1D15 as a key effector of mitochondria-linked autophagy and suggest potential therapeutic avenues for disorders with defective PINK1/Parkin mitophagy [39,40], while highlighting the need for further in vivo and mechanistic studies.

## Figures and Tables

**Figure A1:**
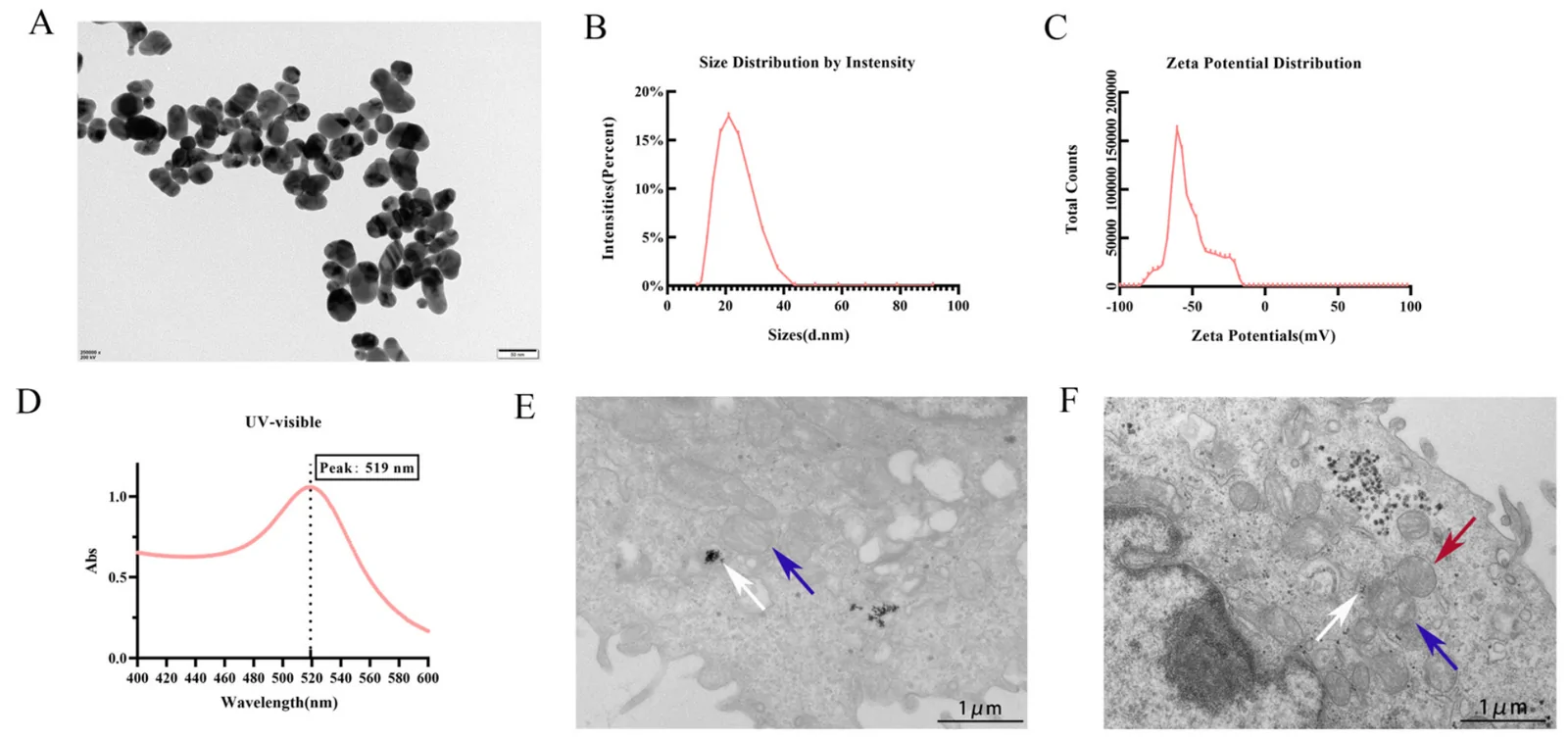
Physicochemical characterization of gold nanoparticles (AuNPs) and TEM ultrastructural observation of AuNP−triggered mitochondria–lysosome interplay. (**A**) TEM micrograph of purified AuNPs. (**B**) DLS intensity−based hydrodynamic size distribution of AuNPs. (**C**) Zeta potential distribution of AuNP suspension. (**D**) UV–visible absorption spectrum of AuNPs with characteristic LSPR peak at 519 nm. (**E**,**F**) Cellular TEM images of AuNP−treated cells. White arrows: intracellular AuNP aggregates; blue arrows: mitochondria; red arrows: lysosomes.

**Figure A2:**
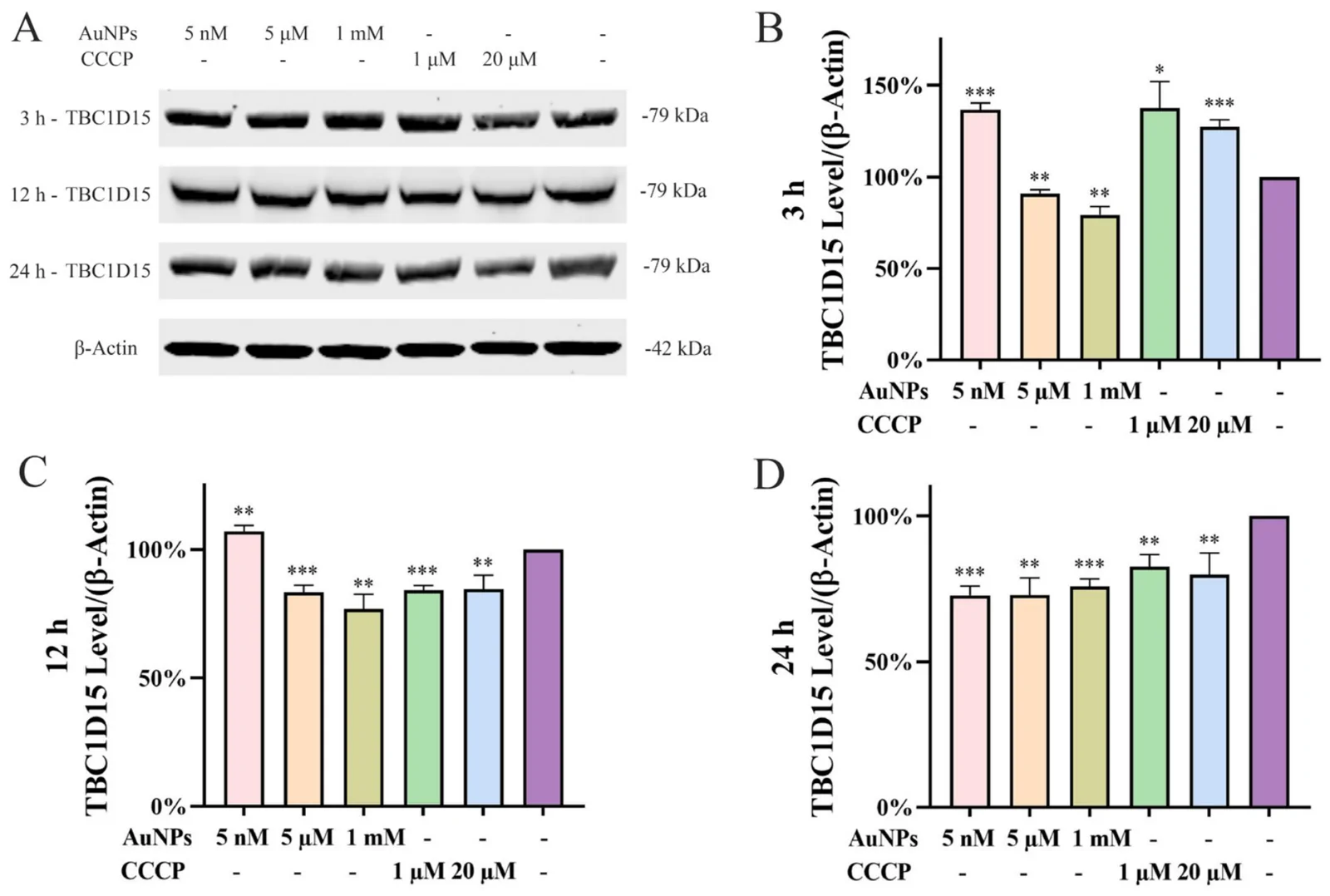
Western blot analysis of TBC1D15 in PC−12 cells. (**A**) Western blot analysis of TBC1D15 protein levels in PC−12 cells treated with AuNPs (5 nM, 5 μM, 1 mM) or CCCP (1 μM, 20 μM) for 3, 12, and 24 h. β−Actin served as the loading control. (**B**) Quantitative analysis of TBC1D15/β−Actin ratios after 3 h treatment. (**C**) Corresponding ratios after 12 h treatment. (**D**) Corresponding ratios after 24 h treatment. Statistical significance: * *p* ≤ 0.05, ** *p* ≤ 0.01, *** *p* ≤ 0.001 (*n* = 3).

**Figure A3:**
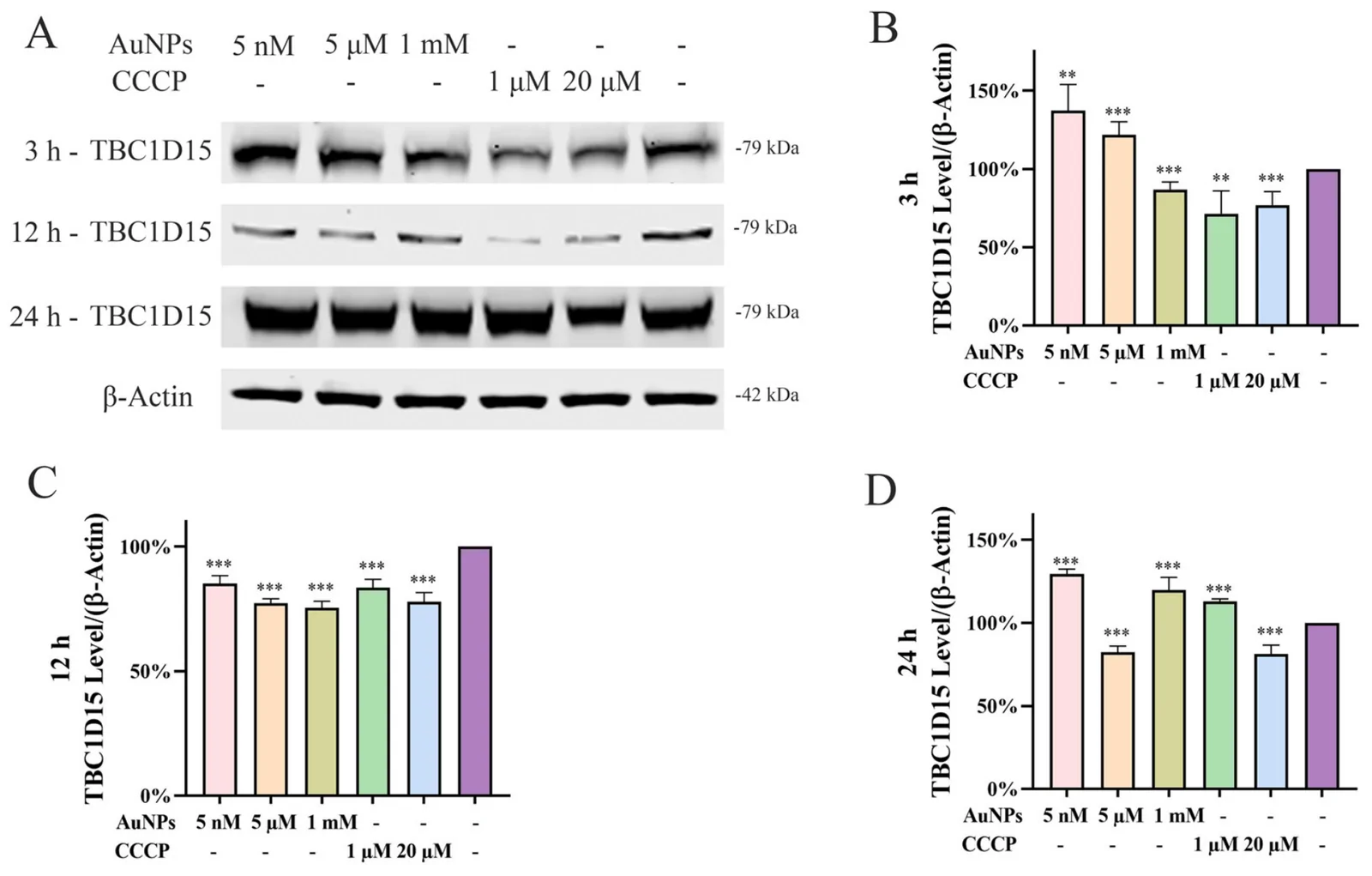
Western blot analysis of TBC1D15 in HeLa cells. (**A**) Western blot analysis of TBC1D15 protein levels in HeLa cells treated with AuNPs (5 nM, 5 μM, 1 mM) or CCCP (1 μM, 20 μM) for 3, 12, and 24 h. β−Actin served as the loading control. (**B**) Quantitative analysis of TBC1D15/β−Actin ratios after 3 h treatment. (**C**) Corresponding ratios after 12 h treatment. (**D**) Corresponding ratios after 24 h treatment. Statistical significance: ** *p* ≤ 0.01, *** *p* ≤ 0.001 (*n* = 3).

**Figure A4:**
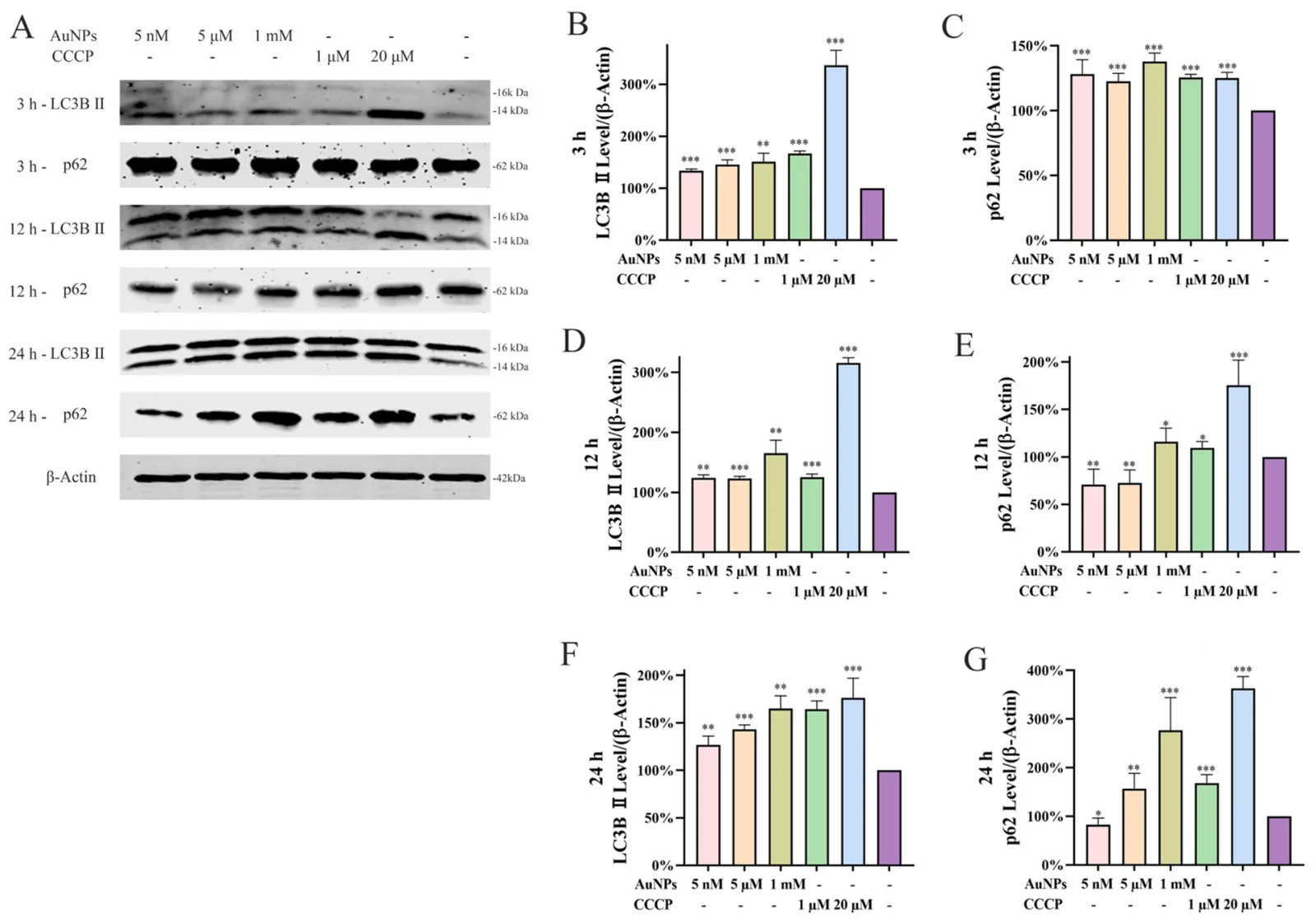
Western blot analysis of LC3B−II and p62 in PC−12 cells. (**A**) Western blot analysis of LC3B−II and p62 protein levels in PC−12 cells treated with AuNPs (5 nM, 5 μM, 1 mM) or CCCP (1 μM, 20 μM) for 3, 12, and 24 h. β−Actin served as the loading control. (**B**,**C**) Quantitative analysis of LC3B−II/β−Actin and p62/β−Actin ratios after 3 h treatment. (**D**,**E**) Corresponding ratios after 12 h treatment. (**F**,**G**) Corresponding ratios after 24 h treatment. Statistical significance: * *p* ≤ 0.05, ** *p* ≤ 0.01, *** *p* ≤ 0.001 (*n* = 3).

**Figure A5:**
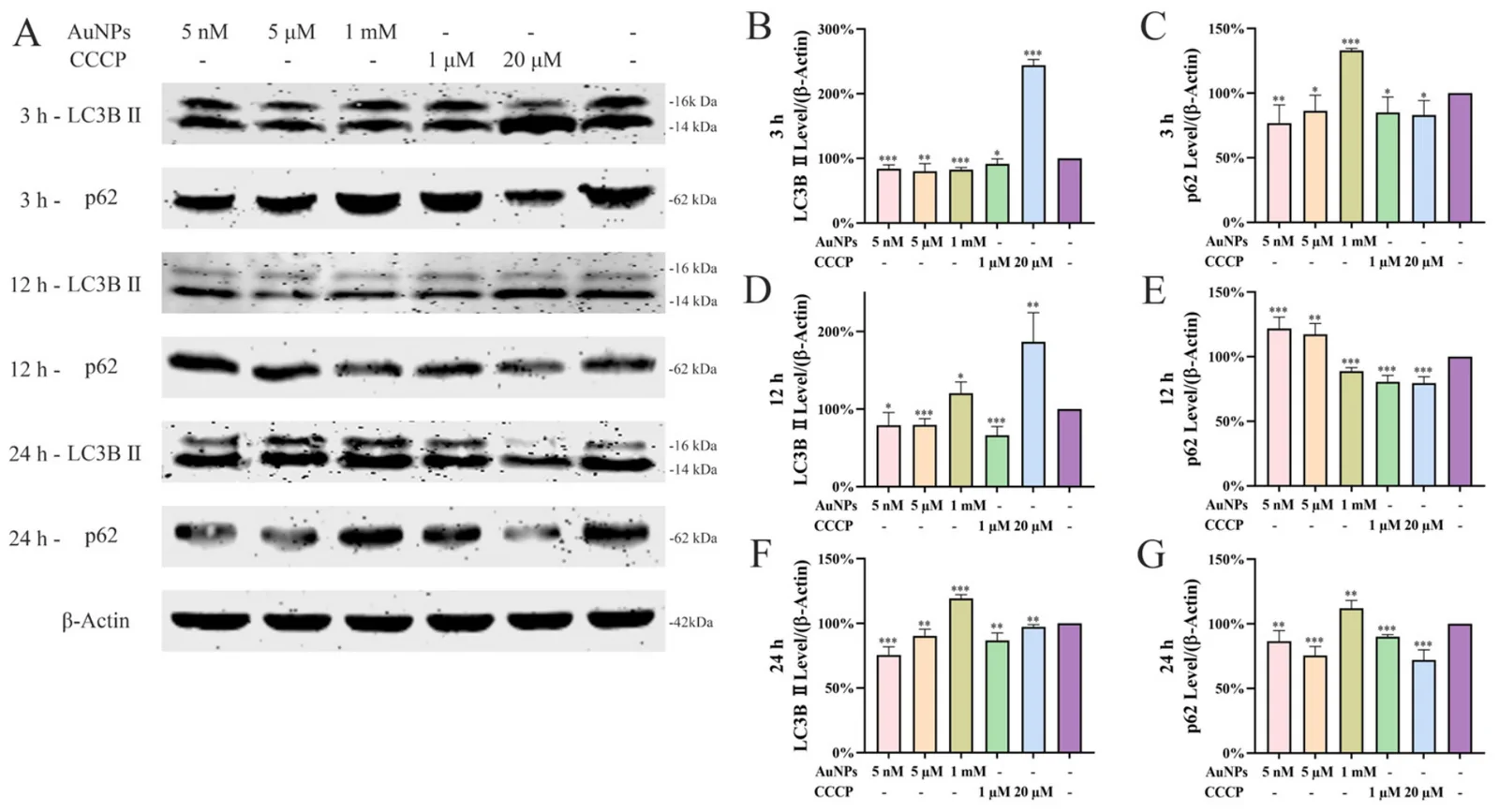
Western blot analysis of LC3B−II and p62 in HeLa cells. (**A**) Western blot analysis of LC3B−II and p62 protein levels in HeLa cells treated with AuNPs (5 nM, 5 μM, 1 mM) or CCCP (1 μM, 20 μM) for 3, 12, and 24 h. β−Actin served as the loading control. (**B**,**C**) Quantitative analysis of LC3B−II/β−Actin and p62/β−Actin ratios after 3 h treatment. (**D**,**E**) Corresponding ratios after 12 h treatment. (**F**,**G**) Corresponding ratios after 24 h treatment. Statistical significance: * *p* ≤ 0.05, ** *p* ≤ 0.01, *** *p* ≤ 0.001 (*n* = 3).

## Data Availability

All data reported in this paper will be shared by ScienceDB, Http://Doi.Org/10.57760/Sciencedb.39624.

## Supplementary Materials

The following supporting information can be downloaded at: https://www.mdpi.com/article/10.3390/cells15191781/s1, Figure S1: Densitometric quantification of LC3B-I/β-Actin ratios in PC-12 cell lines; Figure S2: Densitometric quantification of LC3B-II/β-Actin ratios in PC-12 cell lines; Figure S3: Densitometric quantification of LC3B-I/β-Actin ratios in HeLa cell lines; Figure S4: Densitometric quantification of LC3B-II/β-Actin ratios in HeLa cell lines; Table S1: Key antibodies, chemicals, and oligonucleotides used in this study.

## Abbreviations

The following abbreviations are used in this manuscript:

Abbreviation	Full Name
AuNPs	Gold nanoparticles
BSA	Bovine serum albumin
CCCP	Carbonyl cyanide *m*-chlorophenyl hydrazone
CQ	Chloroquine
DLS	Dynamic light scattering
GAP	GTPase-activating protein
GAPDH	Glyceraldehyde 3-phosphate dehydrogenase
HSP60	60 kDa heat shock protein
LAMP1	Lysosomal-associated membrane protein 1
LC3B-II	Microtubule-associated protein 1A/1B-light chain 3B-II
PBS	Phosphate-buffered saline
PINK1	PTEN-induced kinase 1
PMSF	Phenylmethylsulfonyl fluoride
RIPA	Radioimmunoprecipitation assay
SDS-PAGE	Sodium dodecyl sulfate–polyacrylamide gel electrophoresis
siRNA	Small interfering RNA
TBC1D15	TBC1 domain family member 15
TBST	Tris-buffered saline with Tween 20
TEM	Transmission electron microscopy
UV–vis	Ultraviolet–visible

## Appendix A

### Appendix A.1. Characterization of Synthesized Gold Nanoparticles (AuNPs) and Intracellular Organelle Colocalization Induced by AuNP Treatment

The physicochemical properties of the prepared AuNPs were systematically characterized by transmission electron microscopy (TEM), dynamic light scattering (DLS), zeta potential analysis, and UV–vis spectrophotometry (Figure A1). TEM imaging revealed that the as-synthesized AuNPs exhibited uniform quasi-spherical morphology with well-distributed particles and minimal large aggregates. DLS size distribution analysis demonstrated that the intensity-weighted hydrodynamic diameter of AuNPs centered at 23.93 ± 0.44nm, consistent with the particle size of 19.18 ± 5.29 nm that observed under TEM. Zeta potential measurement showed a sharp negative peak centered at −42.1 ± 3.6 mV, indicating that the AuNP surface carried abundant negative charges, which provided strong electrostatic repulsion and guaranteed the colloidal stability of the nanoparticle dispersion. The polydispersity index (PDI) of the AuNP suspension was determined by DLS and found to be 0.14 ± 0.03, indicating a narrow and homogeneous size distribution. UV–visible absorption spectroscopy displayed a characteristic localized surface plasmon resonance (LSPR) absorption peak at 519 nm, a typical optical signature of ~20 nm spherical gold nanoparticles, further verifying the successful synthesis of homogeneous AuNPs.

To visualize the intracellular distribution of AuNPs and their influence on mitochondria–lysosome interplay, cellular ultrastructure was observed via TEM of AuNP-treated HeLa cells. Black electron-dense AuNP aggregates (marked by white arrows) were clearly visible inside cytoplasmic vesicular compartments. Red arrow labels lysosomal structures, while blue arrows indicate mitochondria. In the AuNP-exposed cells, extensive physical contact between mitochondria and lysosomes was frequently captured, demonstrating that AuNP stimulation promotes the formation of mitochondria–lysosome contact sites, which provides direct ultrastructural evidence for AuNP-mediated regulation of organelle crosstalk and mitophagy homeostasis.

### Appendix A.2. Dose- and Time-Dependent Alterations of TBC1D15 upon AuNP and CCCP Exposure in PC-12 and HeLa Cells

To evaluate how AuNPs and CCCP influence TBC1D15 protein expression, we measured its levels following treatment in PC-12 and HeLa cells.

In PC-12 cells, TBC1D15 expression exhibited time- and concentration-dependent changes. After 3 h of treatment, low-concentration AuNPs (5 nM) and CCCP (1 µM and 20 µM) increased TBC1D15 levels relative to untreated controls (Figure A2). By 12 h, TBC1D15 expression decreased in cells treated with higher concentrations of AuNPs (5 µM, 1 mM) or CCCP, whereas it remained elevated under low-concentration AuNPs (5 nM). After 24 h, all treatment groups—including AuNPs (5 nM–1 mM) and CCCP (1–20 µM)—showed reduced TBC1D15 expression compared to controls, albeit to varying degrees.

A similar concentration- and time-dependent pattern was observed in HeLa cells (Figure A3). At 3 h, lower AuNP concentrations (5 nM, 5 µM) increased TBC1D15 levels, while higher AuNPs (1 mM) and CCCP (1 µM, 20 µM) decreased them. By 12 h, all treatments led to reduced TBC1D15 expression. After 24 h, TBC1D15 levels rebounded in cells treated with 5 nM or 1 mM AuNPs and low-concentration CCCP (1 µM), but remained suppressed under 5 µM AuNPs or high-concentration CCCP (20 µM).

### Appendix A.3. Modulation of Autophagic Flux Induced by AuNPs and CCCP via LC3B-II/p62 Detection

We next assessed autophagy activity by monitoring LC3B-II and p62 protein levels in PC-12 and HeLa cells following AuNP or CCCP treatment.

In PC-12 cells, both AuNPs and CCCP generally increased LC3B-II levels across all time points and concentrations tested (Figure A4). At 3 and 12 h, AuNPs and low-concentration CCCP (1 µM) induced comparable LC3B-II elevations, whereas high-concentration CCCP (20 µM) triggered a more pronounced increase. p62 levels rose in all groups at 3 h. By 12 h, p62 decreased under low AuNP concentrations (5 nM, 5 µM) but remained elevated in other conditions. At 24 h, LC3B-II remained elevated across treatments, while p62 decreased only with low-concentration AuNPs (5 nM), increasing in a concentration-dependent manner in all other groups.

In HeLa cells, autophagy markers also displayed time- and concentration- dependent changes (Figure A5). At 3 h, AuNPs and low-concentration CCCP reduced LC3B-II levels, whereas high-concentration CCCP increased them. p62 decreased in all groups except high-concentration AuNPs (1 mM), which induced p62 accumulation. By 12 h, LC3B-II increased only with high-concentration AuNPs or CCCP, and p62 rose only under low AuNP concentrations. After 24 h, LC3B-II remained elevated only with high-concentration AuNPs, and p62 accumulation persisted exclusively in the high-concentration AuNP group.
