# Supplementary material for: Disrupting Mitochondrial Homeostasis via TBC1D15 Protein
Source: Cells. 2026 Sep 30;15(19):1781. doi: 10.3390/cells15191781 (PMC13663936; doi:10.3390/cells15191781)
Supplement: Supplementary file 1 [file cells-15-01781-s001.zip › cells-4566013-supplementary.pdf]

Supplementary Materials

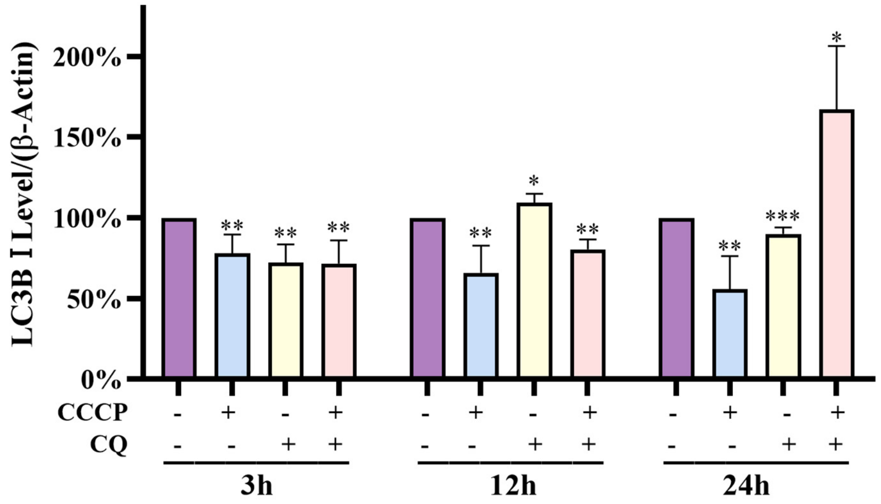

Figure S1. Densitometric quantification of LC3B-I/β-Actin ratios in PC-12 cell lines.

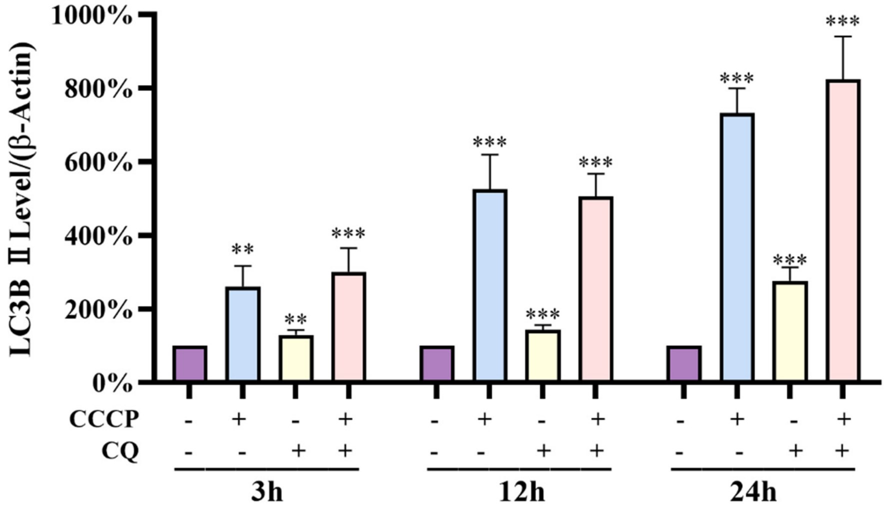

Figure S2. Densitometric quantification of LC3B-II/β-Actin ratios in PC-12 cell lines.

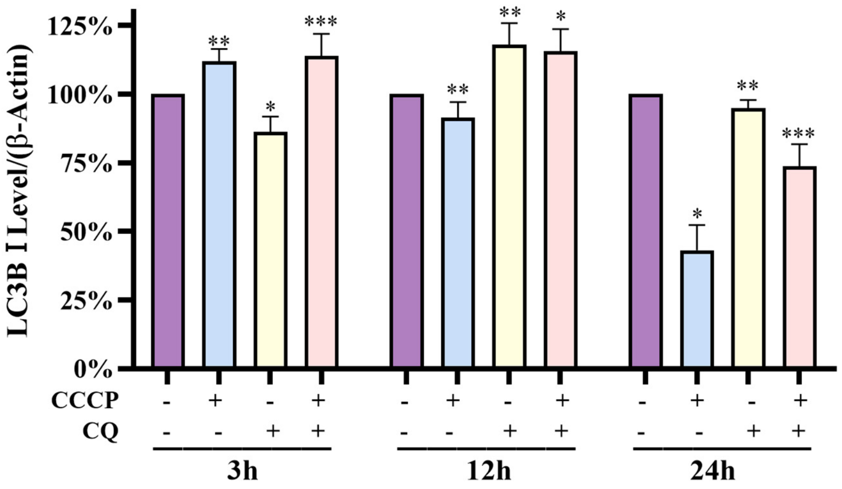

Figure S3. Densitometric quantification of LC3B-I/β-Actin ratios in Hela cell lines.

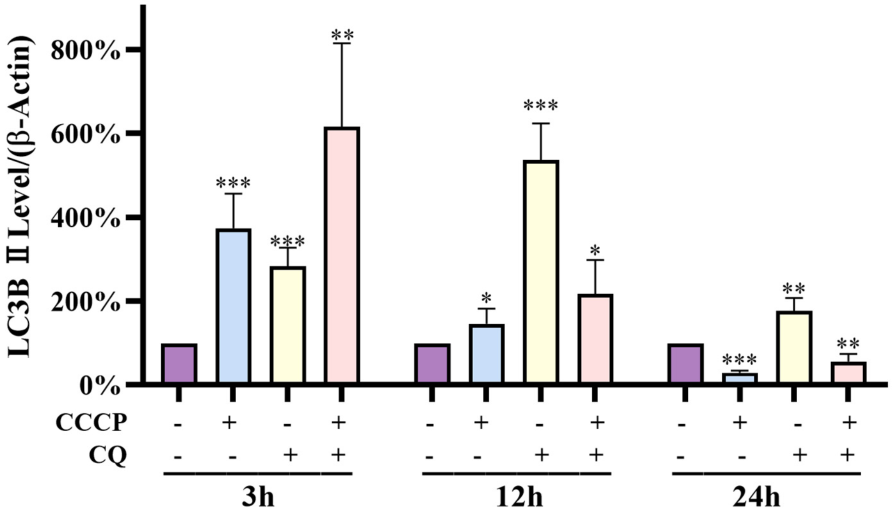

Figure S4. Densitometric quantification of LC3B-II/β-Actin ratios in Hela cell lines.

Table S1. Key antibodies, chemicals, and oligonucleotides used in this study.

REAGENT or RESOURCE	SOURCE	IDENTIFIER	REMARK
	Antibodies		
IRDye® 800CW Goat anti-Rabbit IgG Secondary Antibody	LI-COR Biosciences	Selected P/N: 926-32211	Work dilution: 1:10000
Anti-PINK1 antibody produced in rabbit	Sigma-Aldrich Corporation	P0076 54080501	Work dilution: 1:750
TBC1D15 Polyclonal antibody	Abcam plc	PA5-5305 RRID:AB_2648252	Work dilution: 1:250
LC3B Antibody	Cell Signaling Technology	#2775	Work dilution: 1:1000
SQSTM1/p62 Rabbit mAb	ABclonal Technology Co., Ltd	A19700	Work dilution: 1:10000
β-Actin Rabbit mAb	ABclonal Technology Co., Ltd	AC026	Work dilution: 1:100000
GAPDH Rabbit mAb	ABclonal Technology Co., Ltd	A19056	Work dilution: 1:50000
LAMP1 Monoclonal Antibody	Sigma-Aldrich Corporation	SAB3500285	Work dilution: 1:200
HSP60 Rabbit pAb	ABclonal Technology Co., Ltd	A0564	Work dilution: 1:500
	Chemicals, peptides, and recombinant proteins		
Lipofectamine RNAiMAX reagent	Thermo Fisher Scientific	Product No. 13778030	
Opti-MEM	Thermo Fisher Scientific	31985070	
	Oligonucleotides		
siRNA targeting sequence: PINK1 #1: UUUGCUGAACCCAAGGCUU	Sangon Biotech (Shanghai) Co., Ltd	Lot No:1143852638	
siRNA targeting sequence: PINK1 #2: GGUGUUUGGCCUUCUCCAG	Sangon Biotech (Shanghai) Co., Ltd	Lot No:1143852635	
siRNA targeting sequence: PINK1 #3: GCAAUUUUUACACAGAAAA	Sangon Biotech (Shanghai) Co., Ltd	Lot No:1143852632	
siRNA targeting sequence: PINK1 #4: CCGCAAAUGUGCUUCAUCU	Sangon Biotech (Shanghai) Co., Ltd	Lot No:1143852629	
siRNA targeting sequence: PINK1 #5: CCUCGUUAUGAAGAACUAU	Sangon Biotech (Shanghai) Co., Ltd	Lot No:1143852626	
siRNA targeting sequence: PINK1 #6: GUCACUUACAGAAAAUCCA	Sangon Biotech (Shanghai) Co., Ltd	Lot No:1143852623	
Scrambled siRNA sequence PC-12: CUACGUCAUUGGCAAGCU	Sangon Biotech (Shanghai) Co., Ltd	Lot No:1143852640	
Scrambled siRNA sequence Hela: GCUACGUACUGCAAUCGCU	Sangon Biotech (Shanghai) Co., Ltd	Lot No:1143852631	
